# Combination therapies targeting AD risk factors delay cognitive decline over 10 years in Alzheimer’s patient

**DOI:** 10.1002/alz.093134

**Published:** 2025-01-09

**Authors:** Georgina Torrandell‐Haro, Yuan Shang, Francesca Vitali, Roberta Diaz Brinton

**Affiliations:** ^1^ University of Arizona, Tucson, AZ USA

## Abstract

**Background:**

Alzheimer’s Disease (AD) is a complex neurodegenerative disease characterized by multiple etiologies that remains without a cure. Diabetes, dyslipidemia, hypertension, and inflammation are well‐known risk factors for AD, and FDA‐approved therapeutics for these conditions have been associated with a reduced risk of developing AD. This study aims to evaluate the impact of diabetes medications (DBMD), lipid‐lowering (LIPL), antihypertensive (AHTN), and non‐steroidal anti‐inflammatory (NSD) therapeutics, alone or combined, on cognitive performance in an AD population.

**Method:**

This study used the National Alzheimer’s Coordinating Center (NACC) database that includes longitudinal records of diagnoses, prescriptions, and cognitive performances of AD patients across the United States. A subset of 7,653 AD patients (mean age 74.1 years old) with mean baseline cognitive scores representative of a state of *mild dementia* was selected from 48,605 NACC participants. Change from baseline scores for Clinical Dementia Rating Sum of Boxes (CDR‐SB) and Mini‐Mental State Examination (MMSE) were compared between non‐treated and treated patients over 10 years. Differences in cognitive performance were also evaluated based on sex and APOE genotype.

**Result:**

The quadruplet combination of DBMD+LIPL+AHTN+NSD resulted in a 44% and 35% (MMSE/CDR‐SB) delay in cognitive decline at 5 years and 47% and 35% (MMSE/CDR‐SB) delay at 10 years. The cognitive performance of AD patients treated with monotherapy of LIPL, AHTN, or NSD as assessed by MMSE and CDR‐SB was significantly delayed (10% to 24%) compared to untreated patients at 10 years. Moreover, the data indicated additive and synergistic efficacy in slowing the rate of cognitive decline with combination therapy where slower cognitive decline was observed in patients treated with an incremental number of medications. Moreover, our findings revealed sex‐ and APOE genotype‐specific impact on cognitive performance for each treatment combination, where females and APOE4 exhibited the greatest cognitive benefit of combination therapy.

**Conclusion:**

Combination therapies targeting AD risk factors significantly slowed cognitive decline in AD patients at a magnitude comparable to or greater than beta‐amyloid immunomodulator interventions. Our findings strongly support combination precision medicine, tailored to address multiple AD risk factors within specific subpopulations based on sex and APOE genotype.

